# Fox sightings in a city are related to certain land use classes and sociodemographics: results from a citizen science project

**DOI:** 10.1186/s12898-018-0207-7

**Published:** 2018-11-29

**Authors:** Theresa Walter, Richard Zink, Gregor Laaha, Johann G. Zaller, Florian Heigl

**Affiliations:** 1 Research Institute of Wildlife Ecology, University of Veterinary Medicine, Vienna, Savoyenstrasse 1, 1160 Vienna, Austria; 2 Austrian Ornithological Centre, Konrad Lorenz Institute of Ethology, University of Veterinary Medicine, Vienna, Savoyenstrasse 1a, 1160 Vienna, Austria; 3 Institute for Applied Statistics and Computing, University of Natural Resources and Life Sciences, Vienna, Peter Jordan-Strasse 82, 1190 Vienna, Austria; 4 Institute of Zoology, University of Natural Resources and Life Sciences, Vienna, Gregor Mendel Strasse 33, 1180 Vienna, Austria

**Keywords:** Public participation, Human–wildlife interaction, Carnivores, *Vulpes vulpes*, Urban ecosystems, Remote sensing

## Abstract

**Background:**

Red foxes (*Vulpes vulpes* L.) have become successful inhabitants of urban areas in recent years. However, our knowledge about the occurrence, distribution and association with land uses of these urban foxes is poor, partly because many favoured habitats are on private properties and therefore hardly accessible to scientists. We assumed that citizen science, i.e. the involvement of the public, could enable researchers to bridge this information gap. We analysed 1179 fox sightings in the city of Vienna, Austria reported via citizen science projects to examine relationships between foxes and the surrounding land use classes as well as sociodemographic parameters.

**Results:**

Conditional probabilities of encountering foxes were substantially higher in gardens, areas with a low building density, parks or squares as compared to agricultural areas, industrial areas or forests. Generalized linear model analyses showed that sociodemographic parameters such as education levels, district area, population density and average household income additionally improved the predictability of fox sightings.

**Conclusions:**

Reports of fox sightings by citizen scientists might help to support the establishment of wildlife management in cities. Additionally, these data could be used to address public health issues in relation with red foxes as they can carry zoonoses that are also dangerous to humans.

**Electronic supplementary material:**

The online version of this article (10.1186/s12898-018-0207-7) contains supplementary material, which is available to authorized users.

## Background

Urban areas are increasing worldwide [1], hence, they will become more important for wildlife, especially for small and middle-sized carnivores [2]. For wildlife various potential habitats exist in urban areas including buildings, streets, squares, gardens, parks and other areas all associated with a wide variety of disturbances by humans. These land use classes are used by wildlife in different ways e.g. gardens as resource of food, parks as hiding places or streets for migration. The red fox (*Vulpes vulpes* L., 1758) is one of the globally most adaptive and widely distributed carnivore species [2]. Until the 1980s, foxes in urban areas were mainly reported from the United Kingdom, however, since 1985 they are frequently noticed in many other cities in Canada, Australia, Switzerland, Germany, Japan and Austria [2–4, 25, 62]. There are several reasons why urban areas are attractive to foxes [2]. First, there is a high and constant food availability in urban areas. Second, cities provide safety from interspecific competition. Third, urban areas can provide more shelter or den sites compared to rural areas. Fourth, in cities foxes are safe from being hunted as legal regulations usually restrict hunting near houses [5].

Studying fox ecology in urban areas with focus on occurrence, distribution and use of different land use classes is especially important for public authorities when identifying possible risks of human–wildlife conflicts or disease outbreaks. However, despite the increasing trend in urban fox populations there is still little known about associations between foxes and surrounding land use classes. The challenges to study urban foxes with common non-invasive monitoring methods like camera trapping, transect sampling or hair sampling are great due to (i) omnipresence of people and dogs, (ii) risk of theft of the exposed research equipment in the public space [6–8] and (iii) private property and therefore no access for scientists to many urban habitats frequently favoured by animals (e.g., private gardens, industrial areas) [6, 7, 9]. Findings from studies dealing with urban foxes show that home ranges of urban foxes tend to be smaller than home ranges of rural foxes [10] and urban foxes have been shown to being less territorial and more living in family groups than rural foxes due to more stable food abundances (e.g., Bristol 37 individuals/km^2^ [11], Melbourne 16 individuals/km^2^ [12]; in comparison rural Britain 0.16 to 2.62 individuals/km^2^ [13], rural Germany 0.7 to 2.7 individuals/km^2^ [14]). For the city of Zurich, Switzerland, analysis of fox stomach contents showed that more than 50% of an average stomach content was from anthropogenic food resources [15].

The objective of the current study was to test whether a citizen science approach might be suitable to address the above-mentioned challenges regarding research on urban foxes [6, 9, 16]. We define citizen science as scientific research carried out with the aid of interested volunteers [17]. We are aware of the concerns regarding potential biases of citizen science data regarding geographical coverage and data quality. However, in wildlife research, citizen science has a long-standing tradition and is in the meanwhile also scientifically acknowledged [6, 8, 17]. Despite potential bias in geographic coverage due to unbalanced numbers of participants in some regions of a project or uncertainties in data quality when appropriate quality controls are missing when using citizen science, citizen science can be cost-effective when conducting long term monitoring [18]. Only a few studies address human–carnivore encounters in urban areas [9, 19, 20]. These studies found that wildlife sightings depend on habitat use and activity patterns of wildlife, but also on the use and accessibility of different land use classes by humans, and the visibility of wildlife in different habitats [19, 20]. It was shown by comparing radio-telemetry data and public sightings of urban coyotes that public sightings overestimated the use of more open vegetation as habitat compared to forests with short sight distances. Public sightings were biased towards habitats where people concentrated and daylight when people are more active, although coyotes were moving greater distances at night [19]. The positive association of coyote encounters with building densities in another study was due to more people being present in these areas and not due to coyotes using these areas more frequently [20]. Additionally, both humans and wildlife show certain activity patterns in their daily life, which may influence wildlife sightings [21].

The aim of the current study was to assess to what extent sightings of urban foxes by citizens are influenced by the surrounding land use and/or sociodemographic parameters. In this study, we define sightings as human–fox encounters which are reported via our citizen science project website. Additionally, we investigated temporal changes over years, months and daytime in urban fox sightings. To the best of our knowledge, our study is the first to use citizen science as a non-invasive method to study urban fox occurrence and distribution on a large scale and to include sociodemographic data as an explanatory variable [20]. Results should (i) help researchers to establish a large-scale monitoring system for urban areas by using a citizen science method, (ii) inform wildlife managers in urban areas on human–fox-encounters and therefore (iii) lay the foundation for future systems to prevent human–wildlife conflicts as well as spreading of fox-related diseases.

## Results

### Urban fox sightings

**Fig. 1:**
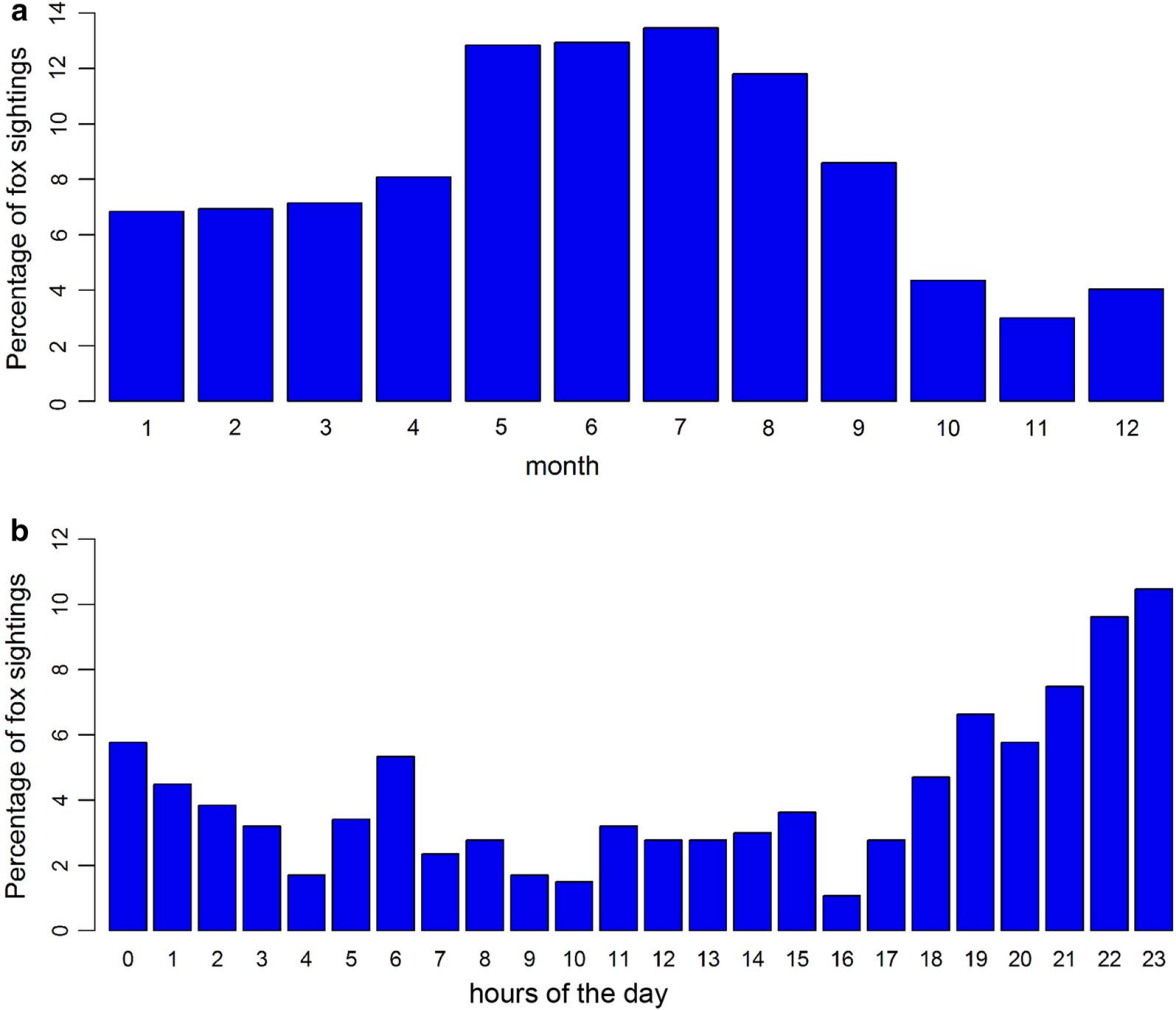
Fox sightings in the city of Vienna per month (n = 966; **a**) and per hour of the day (n = 468; **b**) as percentage of total fox sightings between 2010 and 2015

**Fig. 2:**
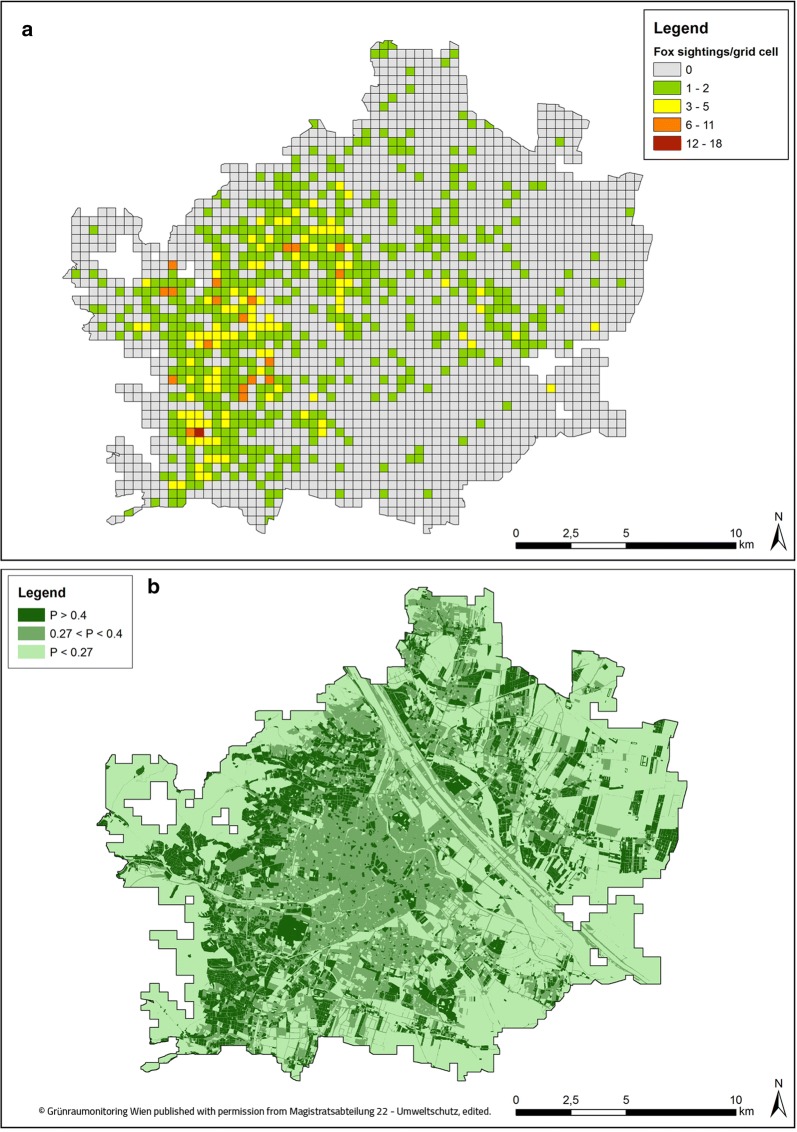
Number of fox sightings in Vienna between 2010 and 2015 per 400 × 400 m grid cell (n = 1179; **a**). Map of conditional probabilities (P) of fox sightings for land use classes in Vienna (**b**). The darker green the area, the higher the values for P

A total of 1179 fox sightings were reported between 2010 and 2015. Foxes were observed in every year of the study duration. The exact date of sighting is known for 966 fox sightings. Fox sightings were not equally distributed across months (Χ^2^ = 171.913, *df* = 11, P < 0.01; Fig. 1a). Across years, most fox sightings were reported in July (n = 130) the fewest in November (n = 29). Foxes in Vienna could be observed at every hour of the day, 41.66% of the sightings were reported between 9 p.m. and 3 a.m. (Fig. 1b). Fox sightings were not equally distributed across the day (X^2^ = 154.2564, *df* = 23, P-value < 0.01). Number of fox sightings per grid cell varied from 0 to 18 (Fig. 2a). The overall probability for sighting a fox in Vienna calculated per grid cell was 0.27. When calculating the conditional probabilities for each land use class, this value was then used as threshold for deciding which land use classes influenced fox sightings positively or negatively.

Conditional probabilities P(E|B) were calculated for all 58 land use classes (Fig. 2b). All land use classes with P > 0.27 are positively associated with fox sightings (Fig. 2b, Table 1), whereas land use classes with P < 0.27 are negatively associated with them (Fig. 2b, Table 2). Land use classes with an increased association of fox sightings accounted for 48.54% and land use classes with a negative association of fox sightings amounted to 51.46% of the total research area. Gardens and areas with a low building density, as well as parks and squares are positive associated with fox sightings, whereas agricultural areas, a diverse range of small other green areas, as well as factory premises and industrial areas and also the forest are negative associated with fox sightings. For some of these land use classes with a negative association of fox sightings, as for instance industrial areas, the relative frequency of fox sightings is rather high compared with others that are positively associated with fox sightings (Tables 1, 2 respectively). However, also the availability of these land use classes is high within the study area, and therefore the probability of fox sightings per land use class is put into perspective by dividing it by *P*(*B*_*j*_), thus calculating the conditional probabilities of fox sightings per land use class.

**Table 1:** Conditional probabilities of fox sightings in Vienna from 2010 to 2015 for all land use classes with a positive association with fox sightings with $P\left( {{\text{E|B}}_{\text{j}} } \right)$ > 0.27

Land use class	$P\left( {E \cap B_{j} } \right)$	$P\left( {B_{j} } \right)$	$P\left( {{\text{E|B}}_{\text{j}} } \right)$
Zoo	0.0003	0.0004	0.892
Pond	0.0003	0.0004	0.647
Leafy property line	0.0003	0.0005	0.555
Park	0.013	0.023	0.544
Square	0.0007	0.001	0.520
Court garden	0.003	0.007	0.503
Flowerbed	0.0007	0.001	0.487
Not developed property (garden)	0.0007	0.002	0.45
Back garden	0.003	0.008	0.45
Terrace, roof garden	0.0001	0.0003	0.419
Detached house garden	0.049	0.118	0.417
Allotment	0.011	0.026	0.416
Parking space	0.0004	0.001	0.406
Grove	0.002	0.005	0.382
Single trees	0.002	0.005	0.382
Green area in residential neighbourhood	0.029	0.08	0.364
Other land uses	0.003	0.007	0.36
Tree line	0.007	0.02	0.358
Council housing patio	0.0004	0.001	0.352
Alley	0.004	0.012	0.35
Other sealed area	0.0005	0.001	0.346
Roof area	0.0002	0.0005	0.345
Courtyard	0.003	0.007	0.345
Roads	0.016	0.048	0.334
Recreation area	0.007	0.021	0.319
Yard	0.017	0.055	0.311
Vineyard	0.006	0.021	0.305
Traffic island	0.004	0.012	0.287

Land use classes in descending order of $P\left( {{\text{E|B}}_{\text{j}} } \right)$; the higher $P\left( {{\text{E|B}}_{\text{j}} } \right)$, the higher the probability of a fox sighting $P\left( {E \cap B_{j} } \right)$ is the relative frequency of fox sightings in land use class $B_{j}$, $P\left( {B_{j} } \right)$ is its share in the study area

**Table 2:** Conditional probabilities of fox sightings for all land use classes with a negative association with fox sightings with $P\left( {{\text{E}}|{\text{B}}_{\text{j}} } \right)$ < 0.27

Land use class	$P\left( {E \cap B_{j} } \right)$	$P\left( {B_{j} } \right)$	$P\left( {{\text{E|B}}_{\text{j}} } \right)$
Sport facility	0.005	0.018	0.264
Mixed green areas	0.005	0.017	0.263
Swimming facility	0.001	0.004	0.261
Pavement café	0.000	0.000	0.255
Fountain	0.000	0.000	0.248
Littoral zone	0.002	0.008	0.248
Scrubs and meadows	0.001	0.005	0.245
Parking space, camping area	0.002	0.008	0.24
Cemetery	0.004	0.015	0.237
Nursery garden	0.000	0.0001	0.237
Standing waterbody	0.004	0.018	0.231
Derelict green space	0.001	0.002	0.228
Front garden	0.001	0.004	0.221
Railway property, rail track	0.005	0.024	0.22
Meadow, shrubs, young stands	0.007	0.03	0.217
Play ground, sport facility	0.001	0.003	0.215
Relay station	0.003	0.013	0.212
Fruit orchard	0.0001	0.0007	0.205
Forest	0.021	0.112	0.189
Industrial area	0.013	0.074	0.178
Fallow land	0.0008	0.005	0.174
Agricultural business and nursery	0.004	0.023	0.169
Non sealed area	0.0001	0.0005	0.142
Unploughed strip	0.0002	0.001	0.136
Stream	0.003	0.02	0.126
Quarry	0.0002	0.001	0.12
Empty lot	0.0001	0.001	0.118
Fields	0.008	0.108	0.077
Windbreak	0.000	0.001	0.016
Noise barrier	0.000	0.0003	0.006
Small yard, green dominated	0.000	0.000	< 0.001
Plant pot	0.000	0.000	< 0.001

Land use classes in descending order of $P\left( {{\text{E|B}}_{\text{j}} } \right)$; the lower $P\left( {{\text{E|B}}_{\text{j}} } \right)$, the lower the probability of a fox sighting. $P\left( {E \cap B_{j} } \right)$ is the relative frequency of fox sightings in land use class $B_{j}$, $P\left( {B_{j} } \right)$ is its share in the study area

### Influencing factors for fox sightings

Influencing factors for fox sightings were analysed with three different generalised linear models. The model GLM1, containing only percentage of land use classes as predictor variables, showed a highly significant positive influence of different kinds of gardens (detached house gardens, court gardens, allotments), parks and squares on fox sightings, as well as the zoo (Additional file 1: Table S1). Fields, streams, industrial areas and sport fields showed a significant negative influence on human–fox encounters. For this model AIC was 4302.3, Cox and Snell R^2^ = 0.3084 and VIF < 1.5 for all coefficients, therefore meeting the criterion of not exceeding a VIF of 10 [22]. The model GLM2 containing only sociodemographic influence factors showed a significant influence of the number of people with different education levels per district on reported fox sightings per grid cell (Additional file 2: Table S2). Number of reports of a fox sighting increased with increasing numbers of people with a university degree per district and decreased with increasing numbers of people with a compulsory education as highest level of education, increasing district area and average household income. Population density had no significant influence on reported fox sightings (AIC = 4661.4, Cox and Snell R^2^ = 0.18, VIF < 2.8 for all coefficients).

In model GLM3 percentage of land use classes per grid cell was combined with sociodemographic influence factors (Table 3). Positive and negative influence of the different factors on reported fox sightings remained nearly the same, however model fit was improved compared to only considering land use information or sociodemographics (AIC = 4089.9, Cox and Snell R^2^ = 0.3701, VIF < 3.7 for all coefficients).

**Table 3:** Model-averaged coefficients of the GLM3 for the factors influencing fox sightings in the city of Vienna containing land use classes and sociodemographic values as explanatory variables

	Estimate	Std. error	z value	Pr(> |z|)
Detached house garden	1.749	1.44e−01	12.161	< 0.001
Edu_compulsory	− 5.55e−05	4.28e−06	–12.952	< 0.001
Park	1.900	2.12e−01	8.957	< 0.001
Fields	− 1.855	3.73e−01	− 4.973	< 0.001
Forest	− 7.73e−01	1.68e−01	− 4.604	< 0.001
Industrial area	− 1.128	3.06e−01	− 3.686	< 0.001
Stream	− 3.795	7.38e−01	− 5.145	< 0.001
Recreation area	3.515	3.94e−01	8.928	< 0.001
Ave_income	− 7.28e−05	1.46e−05	− 4.998	< 0.001
Edu_university	5.2e−05	1.28e−05	4.052	< 0.001
Allotment	1.577	2.93e−01	5.379	< 0.001
Tree row	3.540	1.03	3.436	< 0.001
Zoo	3.151	8.22e−01	3.835	< 0.001
Court garden	1.982	6.65e−01	2.983	0.003
Leafy property line	5.170	1.907	2.711	0.007
Pond	5.999	2.273	2.639	0.008
District_area	− 1.84e−05	1.15e−05	− 1.610	0.107
Single trees	4.032	2.520	1.600	0.11

## Discussion

Research in urban wildlife ecology and human–wildlife interactions in cities becomes more important as more and more people live in urban areas [1, 2]. This is the first study analysing occurrence and distribution of urban fox sightings in relation to land use and sociodemographics in a European city using a citizen science approach. Fox sightings were not equally distributed across the year and over months, 51% of fox sightings were made between May and August. This could be explained by a fox population peak with many young foxes present and gradually starting to explore greater areas in these months [23]. It should also be considered that the internet platform “StadtWildTiere” was launched and promoted in the public at the end of May and citizens in general are more active outdoors in summer months. Fox sightings were also reported for every hour of the day (Fig. 1). Between 6 p.m. and midnight about 45% of all reported human–fox encounters took place, whereas between midnight and 6 a.m. only about 22% of the sightings were reported. This distribution of sightings is most likely a consequence of human behaviour and activity patterns, rather than due to fox activity patterns, since foxes are considered to be mainly active at night [24]. Gloor found that urban foxes in Zurich preferred public parks and other areas closed for humans during the first half of the night and used residential areas more, when human activity was low in these areas in the second half of the night [25]. For foxes in Bristol (UK) it was even shown that they crossed less roads before midnight than after, therefore supposedly adapting their activity patterns to reduce mortality risks by roads and avoiding human activity [26]. Fox sightings were reported throughout all districts of the city. Our analyses that fox sightings are affected by land use classes suggest that foxes prefer certain land use classes [20]. High conditional probabilities were calculated for different types of gardens and areas with a low building density, as well as for parks and squares. Our citizen science data are thus in line with telemetry studies on urban foxes [10, 25]. People also had good access to these land use classes and foxes are well visible, although during the day they tend to rest in vegetative structures [25]. Low conditional probabilities for fox sightings were calculated for agricultural areas, a diverse range of small other green areas, as well as factory premises and industrial areas and also forests. Based on several studies, one would assume that the number of sightings of foxes on these land use classes in Vienna was as high as that of gardens and parks, however several aspects should be considered [27–29]. First, a sighting of a fox on an agricultural field or in the forest within the city borders may not be as special for people, as a sighting in their own garden. Therefore, foxes seen on those land use classes might not be reported as often as foxes seen in gardens or in parks within the city. These results seem to be consistent with other research which found that sampling effort can bias results of citizen science projects [30–32]. Second, visibility of foxes in a forest is likely to be worse than in gardens or parks. Third, access to industrial areas and factory premises was limited to operating hours and to people who have access. There is the possibility, that in our project we might not have had enough citizen scientists with access to these land use classes, thus resulting in low conditional probability values. A special land use class category was the zoo, situated in the Schönbrunn castle grounds: while accounting for only 0.04% of the study area, foxes were reported in two out of three grid cells containing the zoo as land use class, resulting in the highest conditional probability value of all land use classes. Reported fox sightings from the zoo are sightings of a fox family, which is quite famous among Viennese people, roaming the premises of the zoo, and are not animals held in captivity. The last aspect to consider is of course the possibility that no foxes were present in areas with no reported sightings.

Similarly, to the conditional probability results, analyses with GLMs indicated that fox sightings increased with increasing area of private gardens, public parks and squares. This again mirrored habitat use by urban foxes on a large scale like found in various studies on smaller scales using other methods [10, 25, 33, 34]. These land use classes provide easy access for foxes to food resources as well as shelter. Additionally, these land use classes are also preferred by humans, which makes a human–fox-encounter more likely. As mentioned above, these results certainly do not indicate that land use classes with no fox sightings, inhabit no foxes. It might also be that non-reports from these land use classes originated in human perception of the land use classes as ‘not urban’, therefore not worthy to report a fox sighting to a project on urban wildlife. This is similar to other studies which refer to ‘reporting bias’ in citizen science projects [35, 36]. The GLM containing only sociodemographic predictor variables showed that education level is highly significant, which indicates that people with a university degree reported fox sightings more often than people with only a compulsory education. Since citizen science in general is not restricted to higher educated people [37], this result can be interpreted in a way that our project promotion was focused on the target group of people interested in wildlife. However, the result is also in line with previous findings showing that some citizen science projects seem to be more attractive to people with higher education (e.g. [38]). This challenge of reaching a broad target audience to avoid bias in data collection in citizen science projects could be addressed by training observers with different educational backgrounds or reaching a broader audience through different public relation activities. However, the problem of reaching a broad audience is existing in science communication as well, is multi-facetted and can only be solved by many parallel activities by scientists, communicators, politicians and NGOs. As expected, district area showed no significant influence on explaining fox sightings and human population density did not remain within the model after a stepwise AIC was performed.

Data quality is a core issue of every scientific research project, especially when citizen scientists are involved [39–44]. More than 60% of the sightings were submitted by citizen scientists without a photo for proof. Nevertheless, we considered submissions without a photo of the reported fox sighting to be sufficient, as the red fox is a well-known species and not easily confused with any other wildlife species living in Vienna. In citizen science projects, variation in observer quality and variation in sampling effort over time and space often pose challenges for data analysis [41, 45–47]. Including observer characteristics in statistical analysis can account for variation in data sets gathered by citizen scientists [21, 48]. In our study the combination of land use classes and sociodemographic data lead to a better model than just land use classes. When lacking information on the knowledge of every single observer, sociodemographic census data have been shown to be an important source of variation in citizen science data [20]. Additionally, 20% of the fox sightings were made in private gardens and other forms of private properties, which would be hard to access for researchers [9]. Therefore, citizen science proofed to be a feasible method to research urban foxes.

Citizen science adds different research possibilities to mammal monitoring in urban areas compared to more traditional monitoring methods like camera trapping and transect monitoring. A citizen science approach to wildlife monitoring is appropriate when interactions with wildlife are central to the research question [9]. This can be of high interest when working in urban areas, as human–wildlife contact is increased in certain areas of cities [49]. When researching urban wildlife, the success of a citizen science project can be affected by the species studied. The red fox is a charismatic well-known species and therefore a suited study model. However, even for urban rats, a species not liked by many people, citizen science is nowadays considered as a research method [50]. Additionally, a new possibility of comparing data from different cities arises, when data on wildlife sightings is gathered through the same project design as it is currently done within the project “StadtWildTiere” in Zurich (Switzerland), Berlin (Germany) and Vienna (Austria). Our findings could also have implications for wildlife management in cities or public health issues. For red foxes in Central Europe, infection with and zoonotic transmission of the Fox tapeworm *(Echinococcus multilocularis)* is already of interest for urban areas [51–54]. Human–wildlife interactions affect red fox populations as well as predation rate of the infected intermediate hosts of *E. multilocularis* and should therefore be considered in management strategies of this disease [55].

## Conclusions

Despite common reservations against citizen science as a method, our study demonstrated that these can be partly overcome by including sociodemographic factors in the analyses. Taking the results of this study as basis for future citizen science projects in urban areas, we recommend to develop advanced citizen science projects with a broad focus on various target groups to foster the reporting of fox sightings on a large scale. The mostly positive feeling associated with a personal observation of wildlife in the city during a citizen science project could be followed by a more relaxed coexistence of humans and animals in the cities in general. Additionally, such projects would have the potential to predict the likelihood of human–fox encounters in different places of the city to inform public authorities on possible wildlife conflict areas and public health issues.

## Methods

**Fig. 3:**
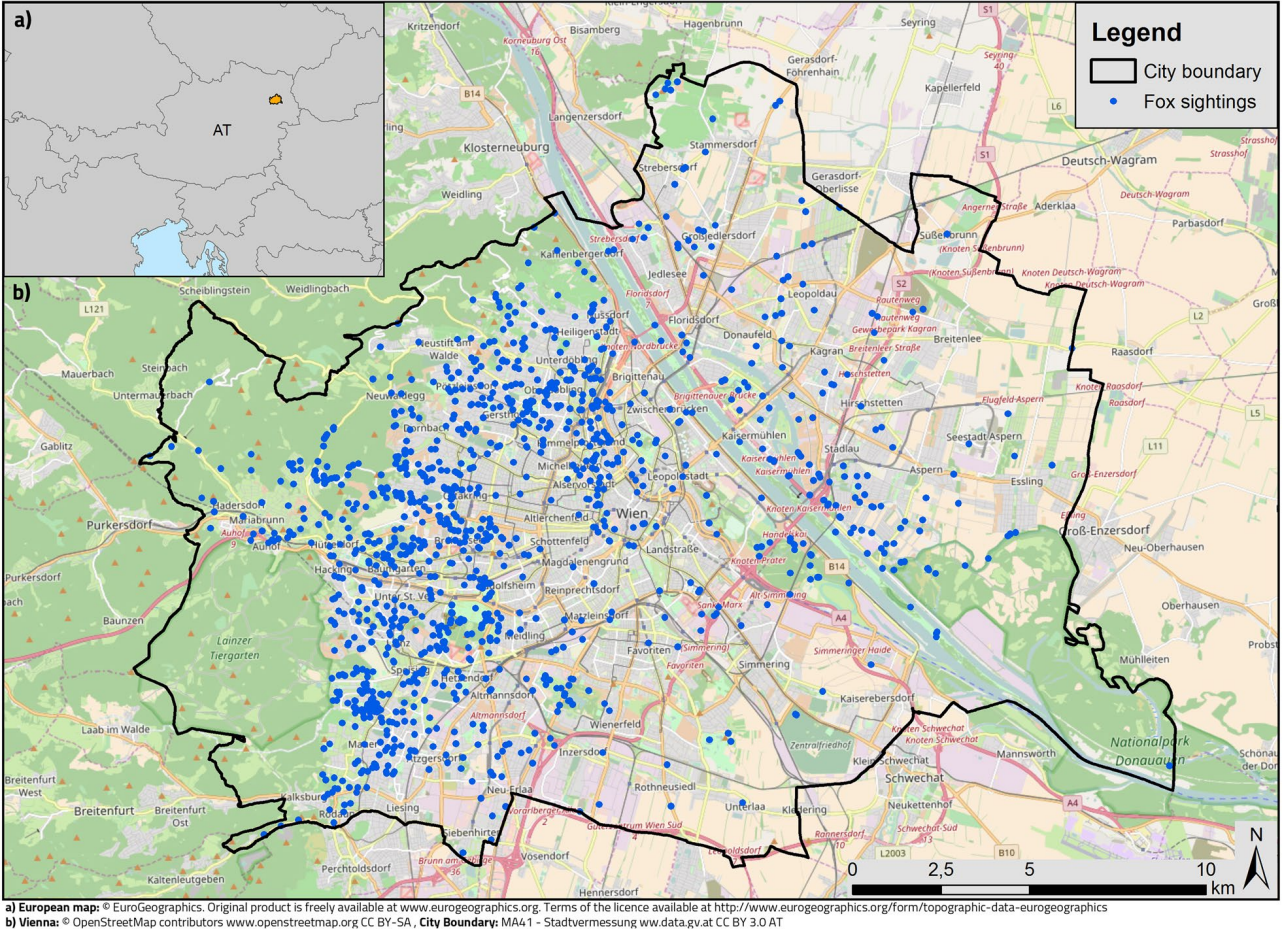
Location of the study area (**a**). Fox sightings reported between 2010 and 2015 (n = 1179; **b**)

The current study uses data from the citizen science project “Wildtiere in Wien” (translated “wildlife in Vienna”) running from 2010 until May 2015, and its follow-up project “StadtWildTiere” (translated “urban wildlife”; http://www.stadtwildtiere.at). The citizen science projects were conducted in Vienna, the capital city of Austria, with a total area of 414.87 km^2^ and about 1.8 million inhabitants in 2015 (Fig. 3). Vienna is surrounded by the Viennese forest in the west, the agricultural plains of the Marchfeld to the northeast, the floodplains of the Lobau to the east, and the Viennese Basin to the south. Green areas (e.g. forests, agricultural areas, parks) make up 45.1% of the city area, 35.8% are building areas, 14.4% traffic areas (e.g. roads, railway tracks), the remaining 4.7% of the area are water bodies. The percentage of green area varies between 2 and 15% in the inner districts and can reach up to 70% in the districts at the fringe of the city. The green spaces are well connected on the city edges and rather patchy in the centre, however a diverse range of rivers and train tracks connects green areas throughout the city [56]. Since the outskirts of Vienna are rural dominated, the actual research area was not defined by the political border of the city of Vienna, but by all buildings in Vienna surrounded by a 400 m buffer within a connected area, resulting in a 36,594 ha research area.

### Data collection and verification

In the first citizen science project “Wildtiere in Wien”, fox sightings were reported by citizens via phone calls, emails, an online questionnaire on the Research Institute of Wildlife Ecology (FIWI) homepage, or at public information days. Through this project 641 sightings were gathered. All of those sightings included information on the species sighted, concerning the date at least the year it was sighted and the location of the sighting. The follow-up project “StadtWildTiere” pursued additional goals and also enabled us to gather more information about wildlife sightings. However, species, date and location are properties of a dataset which were collected in both projects. On the project website citizen scientists are informed about different wildlife species, where animals can be seen, how they can be supported and how conflicts should be managed. The main part of this online platform is designed for easy data entry of wildlife sightings by citizen scientists. Registration is not mandatory to enter data. Different information on sightings is gathered which includes location (either by address or by pinning a point on an implemented Google Map [57]), animal species, number of individuals, date and time of sighting and different kinds of evidence for the presence of an animal (feeding traces, trace mark, scats, den/nest, call, trace marks in snow). Up to three photos can be uploaded for each reported sighting. Location, species and date is mandatory, all other information including photo upload is not mandatory. From 27 May until 15 September 2015, 350 fox sightings were reported via the online form. In addition to citizen science data, we included 188 fox carcasses reported by a service provided by the city of Vienna, which collects animal carcasses (ebs Wien Tierservice, Vienna, Austria*).* After checking all reports for plausibility and deleting entries due to obvious mistakes in data entry, we used 1179 reports of fox sightings for our analyses. From these reports, 29.5% of the sightings were either documented by a photo or a fox carcass that was found; scientists reported 5.7% of all sightings; 64.8% of the sightings were reported without a photo. We considered reports without a photo sufficient, as the red fox is a well-known species not easily confused with any other wildlife species living in Vienna.

### Remote sensing data

The fox sightings were processed using the geographic information system ArcGIS (ESRI ArcGIS 10.2.2) [58]. Vienna’s political borders and district borders were obtained from the platform Open Data Austria [59]. Habitat descriptions were based on 58 land use classes from the official green space monitoring Vienna (Grünraummonitoring Wien). These classes are distinguished due to their vegetation and their potential to serve as a habitat for animals, plants and humans [60, 61]. The land use polygons were intersected with a 400 m × 400 m grid (16 ha) corresponding to the approximate home range of urban foxes calculated for Zurich, Switzerland [25], to obtain land use fractions of possible fox habitats. Zurich was chosen as a reference due to availability of home range data for urban foxes in Central Europe, as well as for similar amount of green spaces in the city and similar lifestyle of people. Overall, the known home ranges of foxes in urban areas vary considerably between seasons, sexes and across the world; individual home ranges range from 5.5 to 70 ha [10, 12, 62].

### Sociodemographic influences

Sociodemographic census data on the population of Vienna provided and collected by the city of Vienna and the Austrian governmental statistics agency “Statistik Austria”, was used to analyse sociodemographic influence factors on fox sightings [63]. Sociodemographic characteristics were available on a district basis. Vienna’s 23 districts have areas ranging from 109 ha to 10,231 ha and a population varying between 16,339 and 189,713 inhabitants [56]. Therefore, each grid cell was assigned to a political district of Vienna according to its position. When grid cells were bordering two or more districts, they were assigned to the district in which most of the grid cell area was located. The sociodemographic characteristics used were: district area (District_area), population density (Population_density), number of people with no more than a compulsory education (Edu_compulsory), number of people with a university degree (Edu_university) and average household income (Ave_income). District area was considered to check for a size-effect, thus whether more foxes would simply be seen in districts with a bigger area. Population density accounted for different degrees of urbanisation (districts with low population density have a more rural character, contrary to highly populated districts with plenty of sealed surfaces) or for an effect of quantity (whether the number of people living in an area would explain the number of fox sightings). The two education levels were included to test whether level of education had an influence on reporting. Finally, a relationship between average household income and vegetation cover in urban areas was suggested by many studies (see [20]), so we tested if the average household income has an influence on fox sightings in Vienna.

### Statistical analyses

For analysing when and where foxes were observed in Vienna the distribution of the fox sightings was analysed according to years, months, and time of day using Chi squared tests.

We subsequently calculated empirical conditional probabilities to analyse the degree to which each land use class is associated with fox sightings [64]. Probabilities of fox sightings on land use classes depend on the size of the area of each land use class. Therefore, it is important to be able to calculate probabilities independently from area sizes. This is done by calculating conditional probabilities through dividing probabilities by the relative share of each land use class in the whole study area. For each land use class B_j_, the i subareas A_ij_ of grid cells with sightings (event E) were determined. The conditional probability P(E|B_j_) of observing a fox on a specific land use class B_j_ is then defined as the sum of areal fractions of class B_j_ of cells with sightings:

$$P\left( {{\text{E|B}}_{\text{j}} } \right) = \frac{{{\text{P}}\left( {{\text{E}} \cap {\text{B}}_{\text{j}} } \right)}}{{{\text{P}}\left( {{\text{B}}_{\text{j}} } \right)}} = \frac{{\mathop \sum \nolimits_{\text{i}} ({\text{A}}_{\text{ij}} /{\text{A}}_{\text{tot}} )}}{{{\text{P}}\left( {{\text{B}}_{\text{j}} } \right)}}$$

where $P\left( {E \cap B_{j} } \right)$ is the relative frequency of fox sightings in land use class $B_{j}$, $P\left( {B_{j} } \right)$ is its share in the study area, $\mathop \sum \nolimits_{i} (A_{ij} /A_{tot} )$ is the sum of areal fractions of class $B_{j}$ in cells with sightings, and $A_{tot}$ the total area under investigation. The conditional probabilities $P\left( {E |B_{j} } \right)$ were finally compared to the overall probability $P\left( E \right)$ of grid cells having a fox sighting in order to conclude which land use classes favor or hamper fox sightings.

To analyze which factors influence fox observations three different generalized linear models (GLMs) with Poisson-distribution were employed [65, 66]. GLM1 had only the percentage of each land use class per grid cell as predictor variables, GLM2 had only sociodemographic values as predictor variables and GLM3 had both percentage of each land use class per grid cell and sociodemographic values as predictors. Stepwise variable selection based on the akaike information criterion (AIC) was used to find the best fitting models. Cox and Snell R^2^ was calculated as generalized coefficient of determination, and variance inflation factors (VIF) were calculated to assess for multicollinearity of predictor variables of each model. As a rule, VIF for all predictor variables in a model should be less than 10 [22]. All analyses were performed using statistical software R 3.2.1 [67].

## Additional files

**Additional file 1: Table S1.** Model-averaged coefficients of the generalised linear model M1 containing only land use classes as explanatory variables that influence fox sightings in the city of Vienna, Austria.

**Additional file 2: Table S2.** Model-averaged coefficients of the generalised linear model M2 containing only sociodemographic values as explanatory variables on fox sightings in the city of Vienna, Austria.

## Abbreviations

AIC: akaike information criterion
FIWI: Research Institute of Wildlife Ecology
GLM: generalized linear models
VIF: variance inflation factors
