# Supplementary material for: Fox sightings in a city are related to certain land use classes and sociodemographics: results from a citizen science project
Source: BMC Ecol. 2018 Nov 29;18:50. doi: 10.1186/s12898-018-0207-7 (PMC6267792; doi:10.1186/s12898-018-0207-7)
Supplement: Supplementary file 1 — Additional file 1: Table S1. Model-averaged coefficients of the generalised linear model M1 containing only land use classes as explanatory variables that influence fox sightings in the city of Vienna, Austria. [file 12898_2018_207_MOESM1_ESM.docx]

**Additional file 1: Table S1** Model-averaged coefficients of the generalised linear model M1 containing only land use classes as explanatory variables that influence fox sightings in the city of Vienna, Austria.

| Land use class | Estimate | Std. Error | z value | P(>\|z\|) |
| --- | --- | --- | --- | --- |
| Detached house garden | 1.903 | 0.141 | 13.535 | < 0.001 |
| Fields | -3.355 | 0.39 | -8.603 | < 0.001 |
| Park | 2.158 | 0.205 | 10.513 | < 0.001 |
| Court garden | 3.694 | 0.633 | 5.838 | < 0.001 |
| Industrial area | -1.735 | 0.322 | -5.391 | < 0.001 |
| Allotment | 1.676 | 0.284 | 5.911 | < 0.001 |
| Tree row | 4.138 | 1.024 | 4.041 | < 0.001 |
| Single trees | 9.797 | 2.417 | 4.054 | < 0.001 |
| Stream | -2.645 | 0.686 | -3.858 | 0.000114 |
| Recreation area | 1.323 | 0.369 | 3.583 | 0.0003 |
| Zoo | 3.681 | 0.817 | 4.507 | 6.58e-06 |
| Leafy property line | 7.378 | 1.979 | 3.729 | 0.0002 |
| Front garden | -6.884 | 2.423 | -2.842 | 0.005 |
| Sports facility | -9.313 | 3.469 | -2.685 | 0.007 |
| Square | 6.984 | 2.538 | 2.752 | 0.006 |
| Pond | 7.012 | 2.277 | 3.079 | 0.002 |
| Forest | -0.333 | 0.164 | -2.032 | 0.042 |
