# Supplementary material for: Fox sightings in a city are related to certain land use classes and sociodemographics: results from a citizen science project
Source: BMC Ecol. 2018 Nov 29;18:50. doi: 10.1186/s12898-018-0207-7 (PMC6267792; doi:10.1186/s12898-018-0207-7)
Supplement: Supplementary file 2 — Additional file 2: Table S2. Model-averaged coefficients of the generalised linear model M2 containing only sociodemographic values as explanatory variables on fox sightings in the city of Vienna, Austria. [file 12898_2018_207_MOESM2_ESM.docx]

**Additional file 2: Table S2** Model-averaged coefficients of the generalised linear model M2 containing only sociodemographic values as explanatory variables on fox sightings in the city of Vienna, Austria

|  | Estimate | Std. Error | z value | P(>\|z\|) |
| --- | --- | --- | --- | --- |
| District_area | -2.65e-05 | 1.15e-05 | -2.304 | 0.021 |
| Population density | 1.19e-06 | 7.35e-06 | 0.162 | 0.871 |
| Edu_compulsory | -5.94e-05 | 4.26e-06 | -13.951 | < 0.001 |
| Edu_university | 4.54e-05 | 1.16e-05 | 3.914 | < 0.001 |
| Ave_income | -3.69e-05 | 1.56e-05 | -2.375 | 0.0175 |
